# Melatonin-Induced Temporal Up-Regulation of Gene Expression Related to Ubiquitin/Proteasome System (UPS) in the Human Malaria Parasite *Plasmodium falciparum*

**DOI:** 10.3390/ijms151222320

**Published:** 2014-12-03

**Authors:** Fernanda C. Koyama, Mauro F. Azevedo, Alexandre Budu, Debopam Chakrabarti, Célia R. S. Garcia

**Affiliations:** 1 Departamento de Parasitologia, Instituto de Ciências Biomédicas, Universidade de São, São Paulo 05508-900, Brazil; E-Mail: fernandakoyama@gmail.com; 2 Departamento de Fisiologia, Instituto de Biociências, Universidade de São Paulo, São Paulo 05508-090, Brazil; E-Mails: maurousp@gmail.com (M.F.A.); alexandre.budu@gmail.com (A.B.); 3 Burnett School of Biomedical Sciences, University of Central Florida, Orlando, FL 32826, USA; E-Mail: dchak@ucf.edu

**Keywords:** melatonin, ubiquitin-proteasome-system, *Plasmodium falciparum*, malaria

## Abstract

There is an increasing understanding that melatonin and the ubiquitin/proteasome system (UPS) interact to regulate multiple cellular functions. Post-translational modifications such as ubiquitination are important modulators of signaling processes, cell cycle and many other cellular functions. Previously, we reported a melatonin-induced upregulation of gene expression related to ubiquitin/proteasome system (UPS) in *Plasmodium falciparum*, the human malaria parasite, and that *P. falciparum* protein kinase 7 influences this process. This implies a role of melatonin, an indolamine, in modulating intraerythrocytic development of the parasite. In this report we demonstrate by qPCR analysis, that melatonin induces gene upregulation in nine out of fourteen genes of the UPS, consisting of the same set of genes previously reported, between 4 to 5 h after melatonin treatment. We demonstrate that melatonin causes a temporally controlled gene expression of UPS members.

## 1. Introduction

*Plasmodium* has a complex life cycle alternating between two hosts (mosquito and vertebrate) undergoing metabolic and morphological changes. In the vertebrate host, merozoites infect red blood cells and continue into growth (ring and trophozoite stages), formation of multiple new merozoites (schizont stage) and finally disruption of red blood cells and releasing of merozoites into the bloodstream that in turn will infect new erythrocytes which is the cause of malaria pathogenesis. This erythrocytic cycle of events usually occur synchronously during *in vivo* infection in *Plasmodium falciparum*, the most virulent of the four *Plasmodium* species that infect humans, and in the rodent malaria parasite *P. chabaudi*. However, synchrony is lost in *in vitro* cultures, presumably because some defining factor present in the host is absent from the culture medium.

Melatonin is well known to have an influence in circadian rhythm [1]. As a pleiotropic molecule, it is found in many organisms such as bacteria, fungi, plants, invertebrates and vertebrates [2,3,4,5]. In humans, melatonin is best known for its production by the pineal gland, it is also synthesized in other cell types including retina, bone marrow, intestinal tract, gonads, and immune system cells [6,7,8,9,10].

Because the *Plasmodium* grows in human host in cycles of 24 h, and the circadian production of melatonin and synchronous growth of *Plasmodium* in human host is a multiple of 24 h, it has been hypothesized that melatonin could be the host factor responsible for *Plasmodium* synchronization *in vivo* [11]. The intraerythrocytic synchronous maturation is lost *in vitro* but melatonin modulates the process by increasing schizont forms of *P. falciparum* and restoring synchronicity of *P. chabaudi* infection of the pinealectomyzed mouse [12]. Remarkably, melatonin is not an exclusive cell cycle modulator of *Plasmodium* species but also has been implicated in *Trypanossoma cruzi* metacyclic differentiation [13] and participates in many host-parasite interactions [14].

The mechanism of melatonin signaling in *Plasmodium* is not clearly understood, but efforts to characterize the pathway revealed the involvement of calcium and cAMP as second messengers in both *P. falciparum* and *P. chabaudi* [15]. In addition, there is an upregulation of the ubiquitin/proteasome system (UPS) genes in *P. falciparum*, and this process is influenced by *P. falciparum* protein kinase 7 [16].

Ubiquitination is a process that adds ubiquitin molecules (a peptide comprised of 79 amino acids) to substrate proteins. Covalent binding of ubiquitin to proteins is currently related to regulation of practically all cellular events in eukaryotes, including the cell cycle, transcription and signal transduction [17].

In general, the ubiquitination occurs in 3 enzymatic steps involving E1 (ubiquitin activating enzyme), E2 (ubiquitin conjugating enzyme), and E3 (ubiquitin ligase) in which the ubiquitin molecule is conjugated to E1 in a thiol-ester bound with consequent adenylation of the *C*-terminal glycine of ubiquitin in an ATP-dependent manner. Subsequently, ubiquitin is transferred to E2 which together with E3, add the ubiquitin to a *C*-terminal ε-amino group of a lysine of the substrate through a covalent isopeptide bond [17]. Polyubiquitinated substrates are then recognized and degraded by 26S proteasome, a multimeric complex comprised of a 20S subunit that possesses the catalytic activity and the 19S regulatory subunit [18].

E3 ubiquitin ligases are mostly responsible for substrate recognition and specificity, acting often in complexes with SCF (SKP1, cullin and F-box) or APC (Anaphase Promoting Complex). This group of enzymes is comprised of protein such as, F-box proteins, U-box proteins, culins, HECT domain (Homologous to E6-AP Carboxyl Terminus) and RING (Really Interesting New Gene). For this reason, compared to E3, the identification of E1 and E2 in genomes is less representative [17].

The deubiquitination process, catalyzed by deubiquitinases, that are cysteine or zinc metalloproteases, removes ubiquitin from substrates. It is noteworthy that the role played by this group of enzymes is currently underexplored, but it is known that mutations in deubiquitinases genes are related to chloroquine and artesunate resistance in *P. chabaudi* [19].

Modulation of UPS by melatonin has also been reported in other organisms. In neuronal cells, melatonin is protective of ischemic brain injury. This neuroprotective effect by melatonin is linked to the upregulation of deubiquitinase protein [20]. In plants, melatonin can alter protein degradation, increasing photosynthetic activity in *Malus hupehensis* [21].

As ubiquitination controls many cell functions including the cell cycle, mainly by leading proteins to proteasomal degradation, the proteasome has also been targeted as an antimalarial [22,23]. Since proteasome inhibitors such as bortezomib are already tested in humans for cancer treatment [24] the administration of such drugs is promising for parasitic diseases control.

The implication of ubiquitination in *Plasmodium* is consistent with the fact that a large number of proteins are ubiquitinated in the parasite’s replicative schizont stage [25]. This suggests that protein degradation is an essential feature for parasite development and/or replication in the red blood cell. Although regulation of cell cycle and ubiquitination are not clearly understood in the human malaria parasite, the participation of UPS in parasite growth and development is in agreement with the analysis of the *P. falciparum* transcriptome, which shows an activation of genes encoding proteasome components in the schizont stage [26]. Moreover an upregulation of the polyubiquitin gene has been reported in late trophozoite and schizont stage, when protein ubiquitination dramatically increases [27].

Although the most studied function of ubiquitination is protein degradation, it is not the only role. In comparison to phosphorylation, ubiquitin chains attached to a protein can be recognized as specific domains (Ubiquitin Binding Domains, UBDs) by diverse effectors. For instance, different lysine chains can form different topologies thus promoting several cellular responses [28]. For example, monoubiquitination is related to gene expression and nuclear transport while polyubiquitination, mainly at lysine 48, encodes for proteasomal degradation [28].

In the work presented here, we characterize gene upregulation in response to melatonin in *P. falciparum* in our effort to characterize the mechanism of melatonin action in the malaria parasite. For this purpose, we assessed time dependent changes in UPS gene expression by qPCR analysis following melatonin treatment and compared these results with our previous analysis [16].

## 2. Results

We have previously reported that UPS gene expression is significantly affected when parasites had been cultured in the presence of melatonin for 5 h [16]. To investigate whether the expression of UPS genes changes in a modular and temporal fashion, a time-dependent analysis was done where parasites were incubated for 3–6 h with the hormone and the transcript levels of UPS genes analyzed every hour.

The criteria for gene selection was the same as in our previous work and consisted of: (I) representative genes from the 3 classes of UPS enzymes *i.e.*, E1, E2 and E3, including E3s from the SCF complex, involved in cell cycle regulation; (II) proteasome subunits and (III) ubiquitin-like proteins (based on [16]).

**Figure 1:**
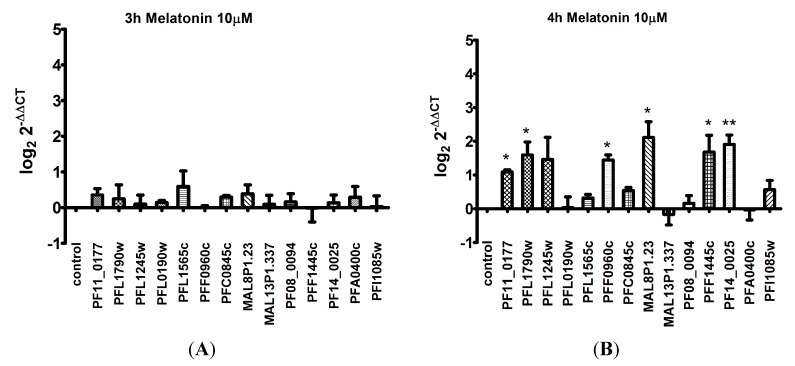

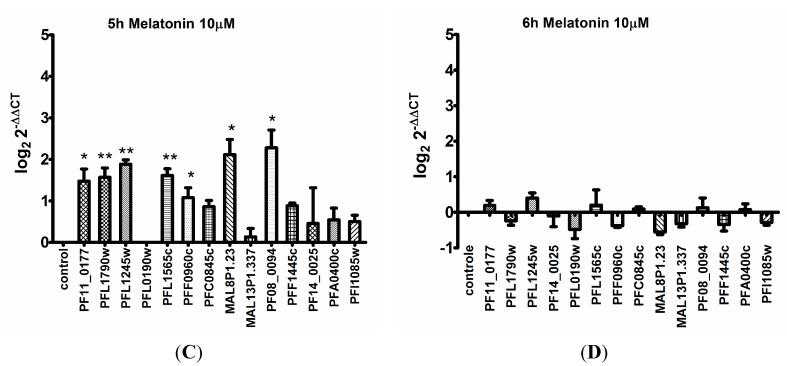
Differential transcription of UPS genes from parasites treated with melatonin treatment. The samples from melatonin treated parasite for 3 to 6 h (**A**–**D**), and that of control cells were compared by real-time PCR analysis. At least three independent experiments were performed (See Table S2). The criterion for altered gene expression was twofold. The ID of the genes is available as supporting information. Statistical analysis was performed with relative expression values in log_2_ by and Student’s *t*-test and Dunnett’s post-test, * *p* < 0.05, ** *p* < 0.01.

As shown in Figure 1 and Table S1, the shortest treatment (3 h) produced no significant changes (Figure 1A). After 4 h of treatment with melatonin, 6 genes were up regulated (Figure 1B, Table S1). The upregulated transcripts are from ubiquitin *C*-terminal hydrolase (PF11_0177), ubiquitin activating enzyme E1 (PFL1790w), hypothetical protein containing the F-box domain (PFF0960c), ubiquitin ligase (MAL8P1.23), culin-like (PFF1445c) and proteasome subunit (PF14_0025). Upregulation of most UPS genes was maintained after 5 h of melatonin, except for proteasome subunit (PF14_0025) and culin-like (PFF1445c) that was no longer significantly elevated (Figure 1C). In contrast, ubiquitin activating enzyme E1 (PFL1245w), a hypothetical protein containing the F-box domain (PFL1565c) and culin-like (PF08_0094) were up regulated only after 5 h of melatonin treatment (Figure 1C). When melatonin treatment was extended to 6 h, expression of all UPS genes returned to levels in between that of control and melatonin treated parasites (Figure 1D). Taken together, these results suggest activation of UPS gene transcription occurs within a short period of 4–5 h following melatonin treatment (Figure 1, Table S1).

##  3. Discussion

*Plasmodium* parasites sense their environment to survive inside red blood cells as evidenced from the transport of cargo within membranous structures to the red blood cell surface and modification of erythrocyte spectrin [29,30]. These changes allow malaria parasites to perceive alterations in their extracellular environment and transduce signals to its subcellular destinations. In this respect, parasite intracellular signaling events are of utmost importance for the successful invasion, development, and egress from red blood cells [31]. Particularly, a complex melatonin/calcium cell signaling network, which involves both classical and novel mechanisms, has been described in *Plasmodium* [12,32,33,34]. This network sheds light on the exploitation of the circadian rhythm of the host by the parasite, leading to parasite intraerythrocytic stage synchronization [12], and in all probability, enhancing parasite survival.

A seminal experiment suggesting that the malaria parasites could employ intracellular calcium for its signaling was performed by Passos and Garcia [35]. By loading permeabilized *Plasmodium* with the membrane-impermeable, fluorescent calcium probe Arsenazo III, it was demonstrated that the parasites possessed a calcium pool which was mobilized by thapsigargin (a SERCA inhibitor, [35]).

Hotta *et al.* [12] demonstrated that *Plasmodium* could perceive the host-produced hormone melatonin, leading to the synchronization of the intraerythrocytic development (*i.e.*, the increase in the percentage of parasites at a particular intraerythocytic stage). Furthermore, it was shown that melatonin was also capable of promoting [Ca^2+^]_cyt_ increase in the parasite, and that a phospholipase C (PLC) inhibitor abrogated both melatonin-induced [Ca^2+^]_cyt_ rise and synchronization, thus linking melatonin to the PLC-calcium intracellular signaling cascade [12]. Furthermore, luzindole, a melatonin receptor antagonist, abrogated both melatonin-induced [Ca^2+^]_cyt_ rise and synchronization [12]. The melatonin precursor *N*-acetylserotonin was also shown to be both permeable to the erythrocyte membrane and capable of modulating the cell cycle of malaria parasites in a Ca^+2^-dependent fashion [32,36]. Further experiments demonstrated that other tryptophan precursors (tryptamine and serotonin) also had the same effect [32]. The classic PLC-generated second-messenger IP_3_ was shown to be produced in a melatonin, dose-dependent fashion in *Plasmodium* [36], and by using caged-IP_3_, released by a UV pulse, melatonin and IP_3_ were shown to possess the same mechanism of calcium mobilization from the endoplasmic reticulum [36].

The mechanism of melatonin action in *Plasmodium* also consists of an intricate cross-talk between calcium and cAMP in both human [15] and murine [33] parasites, in which cAMP evokes [Ca^2+^]_cyt_ rise from the endoplasmic reticulum, which in turn leads to further cAMP rise in the cytosol. In addition, membrane calcium channels are also activated leading to further [Ca^2+^]_cyt_ rise via calcium influx [37].

Mounting evidence suggests the existence of melatonin receptors in *Plasmodium* [12,15,33,36]. Thus, our group conducted a genome-wide search in *Plasmodium* using an *in silico* stratregy, which culminated with the identification of four strong candidates for serpentine receptors in the parasite [38].

Regarding downstream signaling components of the melatonin pathway, it has been demonstrated using fluorescence resonance energy transfer (FRET) peptides as a substrate, that melatonin is capable of enhancing cysteine-protease activity in *Plasmodium* [39]. Furthermore, the transcription factor, PfNF-ҮB, was shown to have its expression and ubiquitination modulated by melatonin [34]. Further experiments are required to better understand how proteolysis and transcription factors are involved in the intraerythrocytic cell cycle regulation of *Plasmodium*.

It has been suggested that melatonin and the ubiquitin/proteasome system seem to interact in order to regulate a variety of cellular processes [40]. In the work presented here, we provide evidence for the melatonin-induced modulation of UPS gene expression in *Plasmodium falciparum*. In addition to our previous reports [16,41], which demonstrated that the expression of UPS genes can be affected by a 5 h treatment with melatonin or *N*-acetyl serotonin (NAS), we demonstrate now that some genes are regulated as early as 4 h following melatonin treatment.

A comparison of different duration of treatment that evaluates UPS regulation was made (Table S2) based on qRT PCR analysis of melatonin (different concentrations and time points) as well as NAS treated samples [41]. Our analysis identified 6 genes that are consistently regulated by both melatonin and NAS; they are: ubiquitin *C*-terminal hydrolase (PF11_0177), E1 ubiquitin activating enzyme (PFL1790w), ubiquitin activating enzyme E1 (PFL1245w), hypothetical protein containing the F-box domain (PFL1565c), culin-like (PFF1445c) and proteasome subunit (PF14_0025).

Concerning the features of melatonin-regulated genes, melatonin appears to control both general protein ubiquitination by modulating E1 enzymes and the proteasomal subunit or more specific genes like deubiquitinases and E3 ligases. The proteasome subunit (PF14_0025) also known as RNP6 is a nonenzymatic component of the 19S proteasome lid, essential for ubiquitinated protein degradation and for both *Plasmodium* and *Trypanosome* survival [42,43].

The timing of activation of UPS gene expression seems to be variable since some genes are upregulated after 4 h of melatonin treatment, while others are upregulated only after 5 h. The mRNA quantification suggests a cascade of UPS genes regulation, which is also observed in the protein ubiquitination profile, and therefore, parasite proteins are differentially ubiquitinated over time in the presence of melatonin. Our previous work shows that UPS gene expression regulation by melatonin is dependent on protein kinase 7 [16]. As *Plasmodium* presents a life cycle of 48 h, a fine control of gene expression is expected. Indeed the transcriptome analysis shows a “just-in-time” process of gene transcription induction only at a time when it is required [26].

Because cells utilize UPS for cell growth and development, the parasite UPS could be exploited as a therapeutic target. Finally, as melatonin and UPS have also been involved in *Trypanosoma cruzi* development [13,44], intervention in such pathways by proteasome inhibitors such as bortezomib could be useful for the control of kinetoplastid protozoan pathogens as well.

## 4. Experimental Section

### 4.1. Parasites

The 3D7 clone of *P. falciparum* was cultured in flasks with Gibco RPMI 1640 medium (Life Technologies, Grand Island, NY, USA) supplemented with 0.04% of gentamicin sulfate, 0.05% of hypoxanthine 0.23% sodium bicarbonate and 10% human serum (blood group A+), and human erythrocytes with a hematocrit of 2%, as previously described in [45]. Parasite cultures were synchronized by the sorbitol treatment which causes a disruption of erythrocytes infected with late trophozoite and schizont [46].

### 4.2. Real-Time PCR of UPS Genes

*P. falciparum* trophozoites were treated with 10 µM melatonin (SIGMA-Aldrich, St. Louis, MO, USA) approximately 24–30 h post invasion. RNA was extracted 3–6 h post treatment with Trizol (Life Technologies) and subjected to DNase treatment. cDNA synthesis was carried out using random primers and Superscript II reverse transcriptase (Life Technologies) according to the manufacturer’s protocol. Oligonucleotides used are listed in Table S1. SYBR green incorporation was measured during PCR amplification performed on the 7300 Real Time PCR System (Applied Biosystems, Foster City, CA, USA). The statistical analysis was performed by applying Student’s *t*-test on the log2 values of relative expression (delta delta *C*_t_ method) [47] of the genes (normalized with the 18 S ribosomal housekeeping gene). The experiments were performed independently at least three times, and each experiment was analyzed in triplicate by real-time PCR.

## 5. Conclusions

Building on our previous work, we have now demonstrated that melatonin elicits activation of UPS gene expression in a time-dependent manner in *P. falciparum*. This study provides evidence of the parasite’s ability to make a fine adjustment of its signalling cascade in order to modulate the parasite erythrocytic developmental cycle.

## Supplementary Materials

Supplementary tables can be found at http://www.mdpi.com/1422-0067/15/12/22320/s1.
